# Measuring the quality of primary care in national health surveys: Lessons from Brazil

**DOI:** 10.4102/phcfm.v12i1.2251

**Published:** 2020-01-29

**Authors:** Erno Harzheim, Luiz Felipe Pinto, Otávio P. D’Avila, Lisiane Hauser

**Affiliations:** 1 Medicine School, Federal University of Rio Grande do Sul, Porto Alegre (UFRGS), Brazil; 2 Department of Medicine in Primary Health Care, Federal University of Rio de Janeiro (UFRJ), Rio de Janeiro, Brazil; 3 Dentistry School, Federal University of Pelotas (UFPel), Rio Grande do Sul, Porto Alegre, Rio Grande do Sul, Porto Alegre, Brazil; 4 Brazilian Health Ministry Consultant, Rio Grande do Sul, Porto Alegre, Brazil

**Keywords:** Africa, Brazil, primary health care, evaluation, PCAT

## Abstract

**Background:**

South Africa started to lead the cross-culturally validation and use of the Primary Care Assessment Tool (PCAT) in Africa, when Professor Bresick filled a gap, as this continent was until then the only one that had never used it in evaluation of primary health care facilities until 2015.

**Aim:**

The authors aim to demonstrate that after the consolidation of Bresick’s team to an African version of PCAT, it had been adapted to household survey in Brazil.

**Methods:**

In this letter, authors reflect on how Brazil had adapted PCAT to a national random household survey with Brazilian National Institute of Geography and Statistics (IBGE) – the Brazilian Census Bureau.

**Results:**

In the the beginning of 2019, Brazilian Ministry of Health brought back the PCAT as the official national primary health care assessment tool. Brazilian National Institute of Geography and Statistics (IBGE) included a new module (set of questions) in its National Health Survey (PNS-2019) and collected more than 100 000 households interviews in about 40% of the country’s municipalities. This module had 25 questions of the Brazilian validated version of the adult reduced PCAT.

**Conclusion:**

We believe that IBGE innovation with the Ministry of Health can encourage South Africa to establish a similar partnership with its National Institute of Statistics (Statistics South Africa) for the country to establish a baseline for future planning of primary health care, for decision-making based on scientific evidence.

## Introduction

### South Africa’s leadership in the use of Primary Care Assessment Tool on the African continent

The questionnaires that form what is known as the Primary Care Assessment Tool (PCAT) were originally created by the team led by Professors Barbara Starfield and Leiyu Shi of Johns Hopkins Bloomberg School of Public Health for the evaluation of essential and derived attributes of primary healthcare services.^1^

In South Africa, between 2015 and 2019, Dr Bresick’s team adapted and cross-culturally validated the PCAT, measured primary healthcare performance in the Western Cape and described differences between the experiences of users and staff with regard to primary care.^2,3,4^ This was the first time that PCAT was used in Africa (the SA PCAT). Since then, another African country, Malawi, has been inspired to follow in the footsteps of Bresick’s team.^5^ The African researchers, just as Brazil did in 2006, when it started to lead and support other Latin American countries in the use of PCAT, could develop partnership with other countries in the continent.

In Brazil, the same questions and domains proposed by Professors Barbara Starfield and Leiyu Shi were maintained in the child and adult PCAT versions.^6,7,8,9^ The new questions in the ZA PCAT help us to reflect on the widening scope of primary healthcare, such as the possibility of referring to a physiotherapist, a mental health professional, a dietician or a social worker.

### Brazilian Institute of Geography and Statistics and the launch of the major national household survey using Primary Care Assessment Tools in the world

Brazil was one of the first Latin American countries visited by Professor Barbara Starfield in 2002, when she launched her Portuguese book on primary healthcare.^10^ She also validated PCAT-Brazil with a team of researchers from southern Brazil and the Ministry of Health to publish it as an instrument for evaluating primary healthcare.^6,11^ The city of Porto Alegre, Rio Grande do Sul, was the first to apply PCAT in Brazil.^6^ Rio de Janeiro, the second major municipality in the country, remains the city in the world that interviewed people the most using the PCAT. In 2014, a group of researchers obtained data from a sample of 6675 users from municipal public health services.^12^

Since 1998, the Brazilian National Institute of Geography and Statistics (IBGE) has been conducting household surveys throughout the country, with probabilistic samples. These surveys, conducted every 5 years, contained a set of questions to investigate the health of the population. Then, in 2013, a new survey – the National Health Survey (Pesquisa Nacional de Saúde – PNS) – contained dozens of modules with specific questions about the health of children, women, the elderly, health service utilisation, etc. Since August 2019, data collection has included more than 100 000 households and about 40% of the country’s municipalities.^13^ One of the modules now has 25 questions from the Brazilian-validated version of the short PCAT^14^ and will be applied to adults aged over 18 years, from August 2019 to February 2020.

This will therefore facilitate the calculation of the general primary care score (the average of all items of the essential and derivative PCAT attributes). It will also allow the comparison of these scores by five regions and 27 federation units

As primary care researchers, we believe that this IBGE innovation with the Ministry of Health can encourage South Africa to establish a similar commitment from its National Institute of Statistics (Statistics South Africa) and the National Demographic and Health Survey to establish a baseline for future planning of primary care and for decision-making based on scientific evidence.
